# Investigation of geometric deformations of the lumbar disc during axial body rotations

**DOI:** 10.1186/s12891-022-05160-9

**Published:** 2022-03-08

**Authors:** Haoxiang Xu, Wangqiang Wen, Zepei Zhang, Jianqiang Bai, Bowen Kou, Jun Miao

**Affiliations:** 1 Clinical Department of Orthopaedics, Tianjin Medical University, Tianjin, China; 2 Department of Spine Surgery, Tianjin Hospital, Jiefangnanlu 406, Hexi District, Tianjin, China

**Keywords:** Lumbar disc, In vivo kinematics, Axial rotation, Weightbearing, Deformation

## Abstract

**Background:**

Quantitative data on in vivo vertebral disc deformations are critical for enhancing our understanding of spinal pathology and improving the design of surgical materials. This study investigated in vivo lumbar intervertebral disc deformations during axial rotations under different load-bearing conditions.

**Methods:**

Twelve healthy subjects (7 males and 5 females) between the ages of 25 and 39 were recruited. Using a combination of a dual fluoroscopic imaging system (DFIS) and CT, the images of L3–5 segments scanned by CT were transformed into three-dimensional models, which matched the instantaneous images of the lumbar spine taken by a double fluorescent X-ray system during axial rotations to reproduce motions. Then, the kinematic data of the compression and shear deformations of the lumbar disc and the coupled bending of the vertebral body were obtained.

**Results:**

Relative to the supine position, the average compression deformation caused by rotation is between + 10% and − 40%, and the shear deformation is between 17 and 50%. Under physiological weightbearing loads, different levels of lumbar discs exhibit similar deformation patterns, and the deformation patterns of left and right rotations are approximately symmetrical. The deformation patterns change significantly under a 10 kg load, with the exception of the L3–4 disc during the right rotation.

**Conclusion:**

The deformation of the lumbar disc was direction-specific and level-specific during axial rotations and was affected by extra weight. These data can provide new insights into the biomechanics of the lumbar spine and optimize the parameters of artificial lumbar spine devices.

## Background

As the most common movement unit, axial rotation movement occurs frequently in daily activities. To obtain the movement characteristics of axial rotation, a large number of in vivo and in vitro studies have been carried out. Recently, cadaver studies have investigated external motion parameters by applying external axial rotational torque and compression loads [1–4]. Many in vivo studies have used imaging techniques to investigate the motion characteristics of different lumbar segments [5, 6]. Based on the kinematic parameters, various finite element models have also been developed to simulate the conditions of the applied load and the rotation of the lumbar spine [7, 8].

However, most of these experiments focused on the contribution of each vertebral body to rotational motion, rather than on the changes in intervertebral disc stress. Recent studies have investigated the deformation parameters of lumbar discs [9–11], with most of the studies focusing on the characteristics of disc deformation during flexion-extension and weightlifting.

In this article, the deformation characteristics of the lumbar disc during axial rotation were analyzed by DFIS technology, which has been widely used in various bone and joint studies. The DFIS builds the human motion model, which determines the relative position of the 3D vertebral body in the software using two orthogonal fluoroscopic images.

A series of experiments were performed to validate the accuracy and repeatability of our equipment. Bai et al. [12] showed that the DFIS had a mean accuracy of less than 0.35 mm and a mean repeatability of 0.36 mm for the image matching technique with experiments of a human lumbar specimen in vivo and a living subject in vitro. The repeatability of the method in reproducing in vivo human spine six degrees of freedom (6DOF) kinematics was less than 0.43 mm in translation and less than 0.65° in rotation.

Specifically, we determined the compression and shear deformations in different areas of the lumbar discs and verified the influence of different loads on the deformation pattern during axial rotation. We assumed that in each region, the deformation of the disc during the axial rotation process was level-dependent and affected by the load.

## Materials and methods

**Fig. 1:**
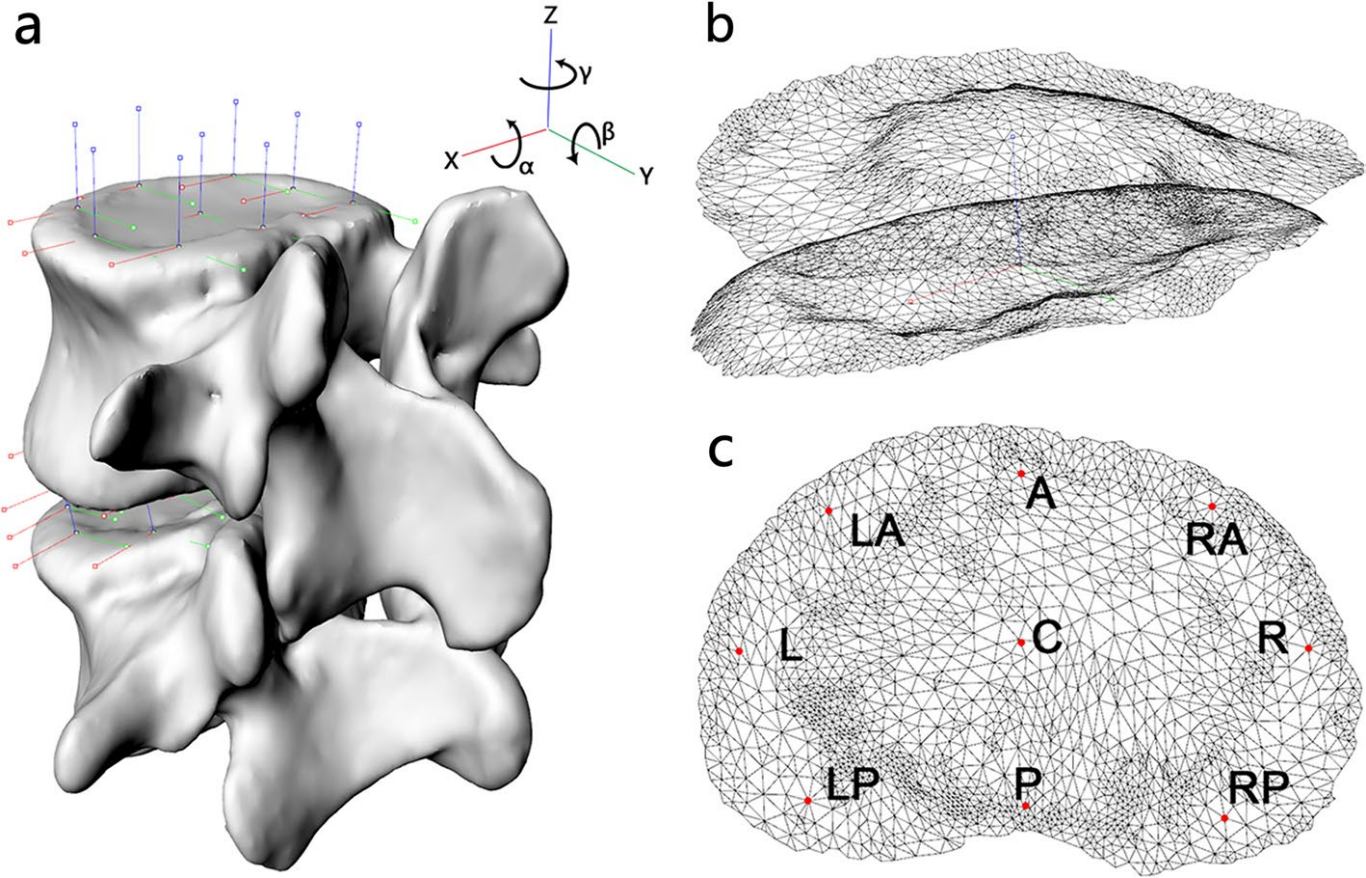
**a** 3D vertebral body with corresponding coordinate system; α-flexion/extension; β-left/right lateral bending; γ-left/right axial rotation; **b** Disc mesh model determined by the adjacent upper and lower endplates; **c** The 9 representative locations on the disc surface. A—anterior, RA—right anterior, R—right, RP—right posterior, P—posterior, LP—left posterior, L—left, LA—left anterior and C—center

Twelve subjects (seven males and five females, ages 25 to 39 years) were recruited. Inclusion criteria included: normal spine development, age 18 ~ 40 years old, BMI 18.5 ~ 25 Kg/m2, Pfirrmann grade ≤ II (MRI), normal bone density. Exclusion criteria included: current or prior low-back pain, previous spinal surgery, anatomic abnormalities, pregnancy, or any spinal disorders. All subjects received a supine CT scan (Sensation 16, Siemens AG, Germany). Parallel digital images of the lumbar spine with a thickness of 0.625 mm and a resolution of 512 × 512 pixels were obtained. These CT images were input into Mimics software (Mimics 19.0 Materialise’s Interactive Medical Image Control System, Belgium) for the construction of 3D models of the vertebrae from L3 to L5 (Fig. 1a). Experienced orthopedic surgeons and radiology specialist excluded 2 subjects with spinal deformities (facet joint disorders) based on CT and 3D models and obtained the final 10 asymptomatic subjects (five males and five females, age 32 ± 4 y, height 1.67 ± 0.09 m, weight 62.75 ± 10.30 kg). The study was approved by our IRB, and signed informed consent was obtained from each subject prior to the experiment. The shape of the lumbar disc was constructed by the three-dimensional volume between the adjacent upper and lower endplates (Fig. 1b). The lumbar disc deformations at the L3–4 and L4–5 segments were investigated, resulting in a total of 20 discs.

A Cartesian coordinate system was created independently for each vertebral body (L3 ~ 5), based on vertebral symmetry (Fig. 1a). In the plane parallel to the upper endplate surface, the x-axis was set to point left, and the y-axis was set to point posteriorly. The z-axis was oriented perpendicular to the x–y plane and pointed proximally [9]. The orientations of the upper vertebral coordinate system in the lower vertebral coordinate system were defined by three rotations using the Euler angles α, β, and γ (in the X–Y–Z sequence): flexion-extension, left-right bending, and left-right axial rotations [13]. The Z-axis difference between the corresponding points of the upper and lower endplates indicates the height of the intervertebral disc. To represent the deformational characteristics of different regions, 9 representative locations on the upper and lower endplates of the discs were chosen: left-anterior, anterior, right-anterior, left, center, right, left-posterior, posterior, and right-posterior (Fig. 1c). The coordinate system was placed at the 9 points and the center of the vertebral body.

**Fig. 2:**
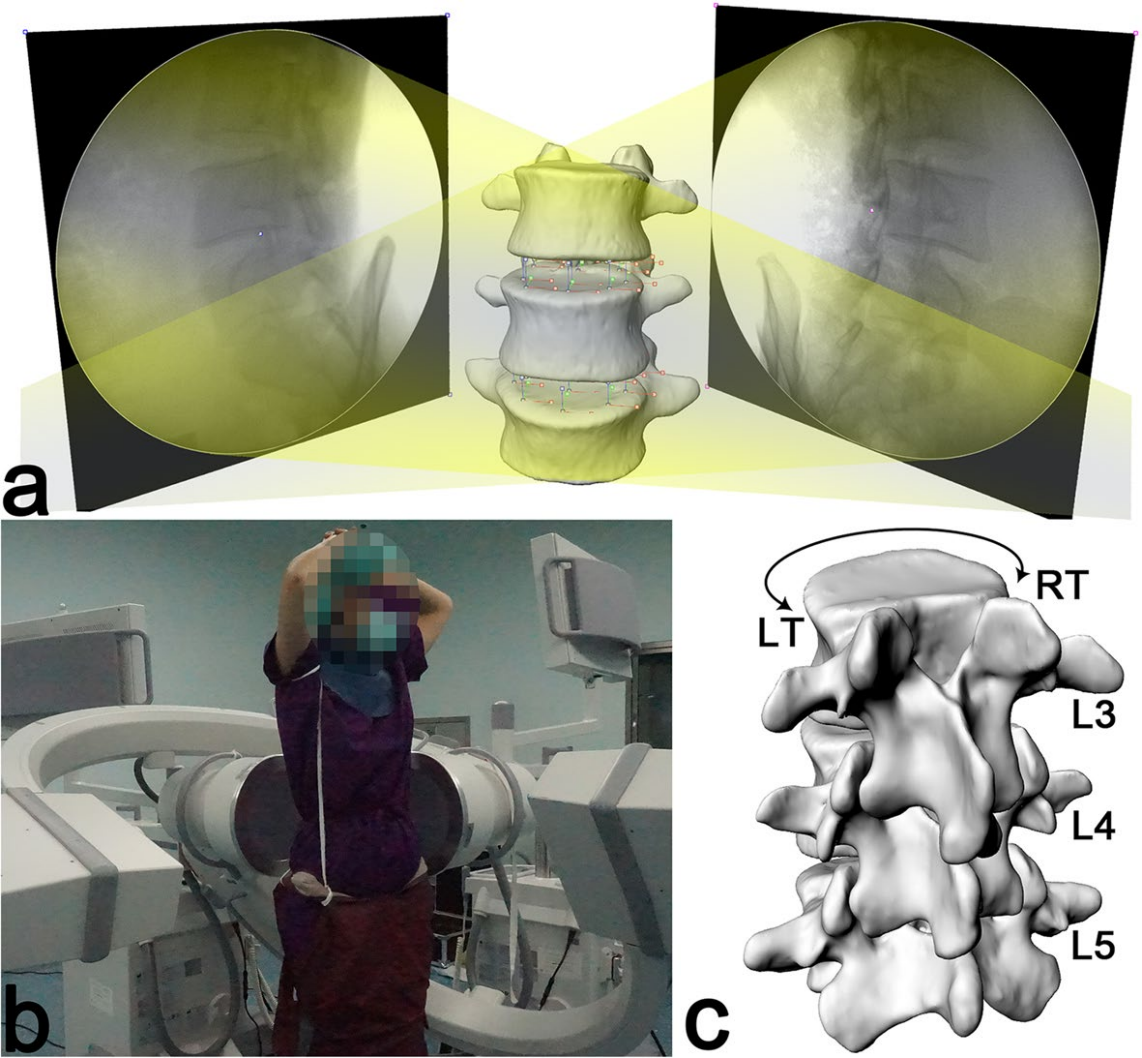
**a** The virtual dual fluoroscopic system that mimics the actual fluoroscopic system, which was used to reproduce the in vivo vertebral positions; **b** Subject protected by custom-made lead clothing; **c** 3D models of the vertebrae from L3 to L5 during left-right axial rotation. LT-left twist, RT-right twist

The lumbar spine of each subject during axial rotations to the maximal left and maximal right in a standing position under weightbearing (0 kg) or a 10 kg load (carrying 5 kg sandbags front and back) was imaged using a dual fluoroscopic imaging system (DFIS). After unified instructional training, the subjects freely rotated their bodies from the standing position to the maximum position and maintained that position for a period of time, during which the researchers assisted in correcting the pelvis and buttocks. Custom-made lead clothing was used to protect the subjects’ thyroid and gonads (Fig. 2a). The two orthogonal fluoroscopic images (F1, F2) obtained at the maximum rotation positions had a resolution of 1024 × 1024 pixels with a pixel size of 0.3 × 0.3 mm^2^ (Fig. 2b).

**Fig. 3:**
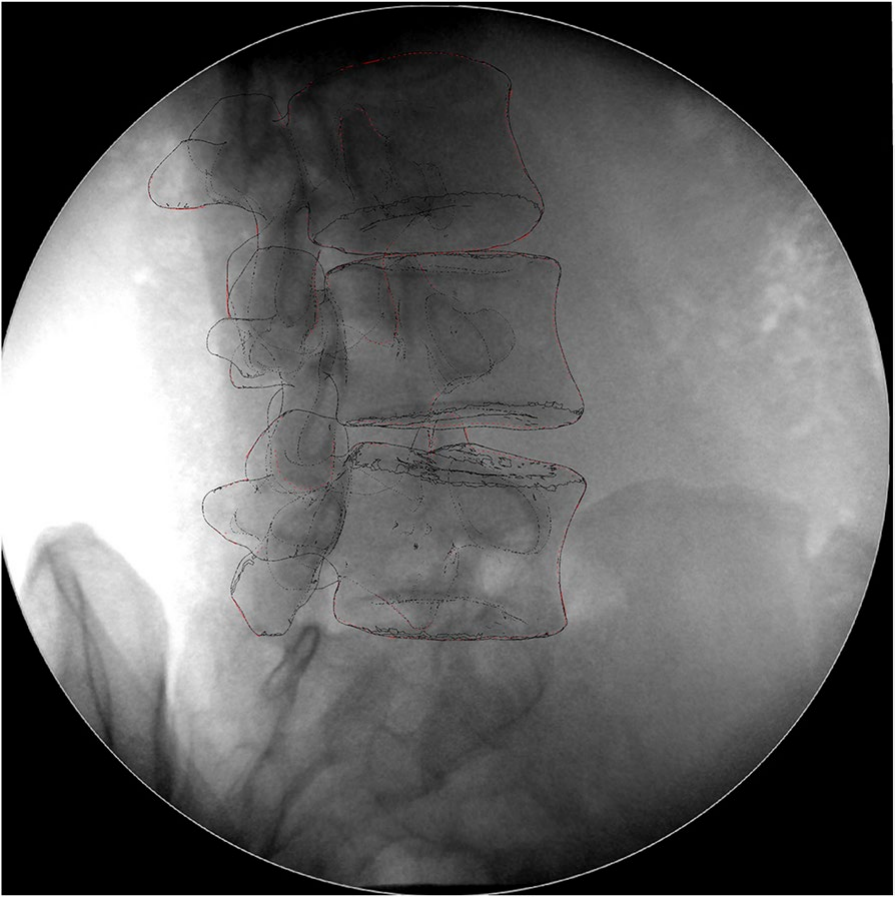
The virtual projection image best matches the paired perspective image in the Rhinoceros software

Using the established protocol, the vertebral model and paired fluoroscopy images were used to reproduce the motion of the vertebral body in Rhinoceros software (version 5.0, Robert McNeel & Associates, United States) [9, 13, 14]. The pairs of fluoroscopic images were imported into the Rhinoceros software environment and rebuilt based on the actual positions of two fluoroscopes to mimic the experimental setup. The environmental files “setup.rvb” were calculated by the disc aligner and the square plate calibrator that was collected before each test. Each 3D model of L3 ~ 5 imported into the DFIS environment produced its own virtual projection images. The in vivo positions of the vertebral body were reproduced when the virtual projection image best matched the pairs of fluoroscopic images in terms of translation and rotation (Fig. 3). Then, the shape of the deformed lumbar disc was determined by the three-dimensional volume between the adjacent upper and lower endplates. The accuracy and repeatability have been validated using a series of experiments [12, 15, 16].

The disc deformation was calculated using mesh vertices evenly distributed on the upper and lower endplates (approximately 3000 points per surface). The coordinate system of the upper endplate surface of the lower vertebra was used as a reference for calculating the displacement of each corresponding point on the lower endplate of the upper vertebra (Fig. 1b). The non-weightbearing, supine position during the CT scan was used as a reference to calculate the shear and compression deformations of each point of the disc during axial rotation motion. Compression deformation represents the change in the disc height, that is, the change ratio of the disc height during axial body rotations to that during supine position. The symbols “+” and “-” mean tensile and compressive, respectively.

$$Tensile\ Deformation=\frac{\mathrm{Disc}\ \mathrm{Height}\left(\mathrm{axial}\ \mathrm{rotation}\right)-\mathrm{Disc}\ \mathrm{Height}\left(\mathrm{supine}\right)}{\mathrm{Disc}\ \mathrm{Height}\left(\mathrm{supine}\right)}$$

$$=\frac{\left|\mathrm{Z}\right|\left(\mathrm{axial}\ \mathrm{rotation}\right)-\left|\mathrm{Z}\right|\left(\mathrm{supine}\right)}{\left|\mathrm{Z}\right|\left(\mathrm{supine}\right)}\left(\%\right)$$

$$\left|\mathrm{Z}\right|=\left|\mathrm{Z}\left(\mathrm{Lower}\ \mathrm{Endplate}\kern0.5em \mathrm{of}\ \mathrm{the}\ \mathrm{Upper}\ \mathrm{Vertebra}\right)-\mathrm{Z}\left(\mathrm{Upper}\ \mathrm{Endplate}\kern0.5em \mathrm{of}\ \mathrm{the}\ \mathrm{Lower}\ \mathrm{Vertebra}\right)\right|$$

+, Tensile Deformation; -,Compressive Deformation. Tension (+) means that the disc height during rotation is greater than that during supine, and compression (−) means that the height during rotation is smaller than that of during supine.

**Fig. 4:**
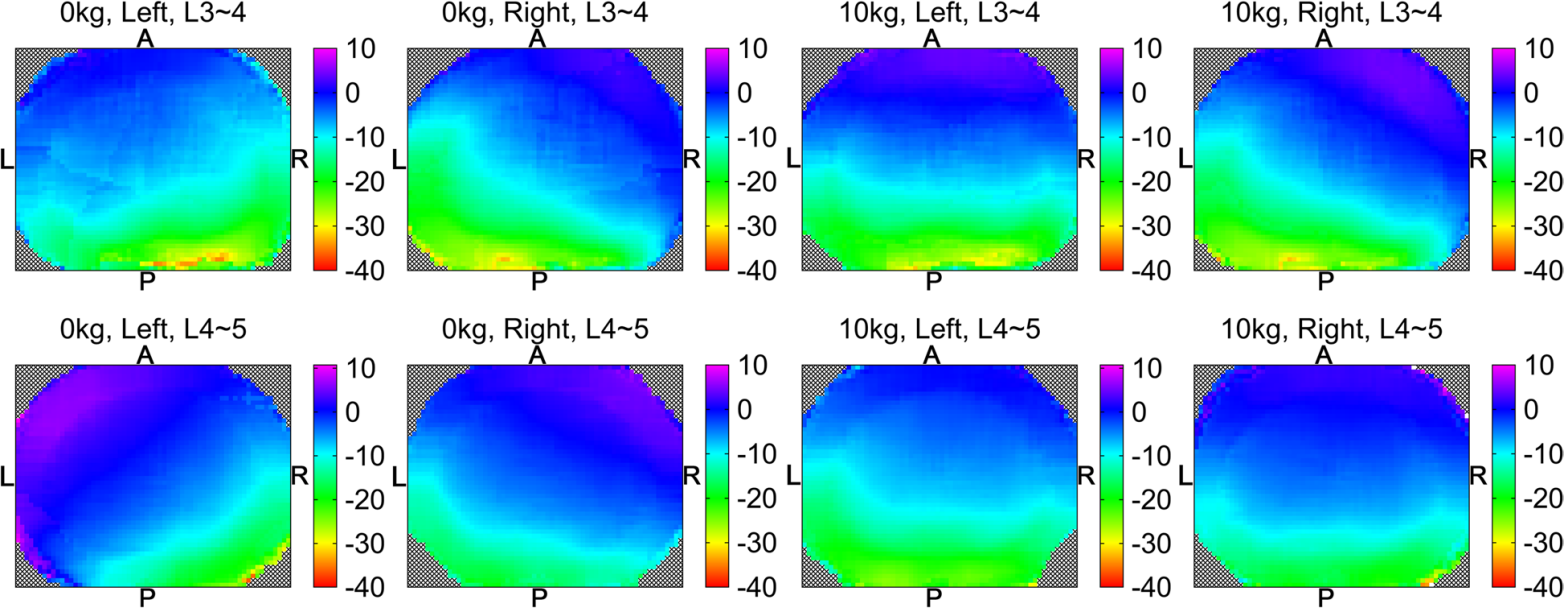
Tensile deformation (%) at different vertebral levels under weightbearing and a 10 kg load. A, anterior; P, posterior; L, left; R, right

The overall compression deformation was measured in the reference coordinate system along the z-axis and plotted on a heat map (Fig. 4). In addition, the overall distributions of shear deformation and compression deformation were analyzed from the average of all subjects based on the 9 representative points.

A two-way analysis of variance was used to compare the differences in shear and tensile deformation, as well as the coupled bending of the lumbar spine during axial rotation motion at different load-bearing levels. The statistical significance was set at *p* < 0.05. Statistical analysis was performed with SPSS 18.0 software (IBM Corp., Armonk, New York).

## Results

### Disc deformation distribution

The overall compression deformation was shown on a heat map (Fig. 4). Going from the CT supine (non-weightbearing) to the standing left rotation (weightbearing) positions, the left-anterior 1/50 of the L3–4 disc area was in tension (+), while the rest of the area of the disc was in compression (−). The magnitude of deformation changed from + 4% tension to − 34% compression in the left-anterior to right-posterior direction. The tendency of tensile and compression deformations during left and right rotations was approximately symmetric. The right-anterior 1/10 of the L3–4 disc was in tension (+) during right rotation. The L4–5 disc under weightbearing had a similar tendency but a larger area of tensile deformation (2/5 during left rotation, 1/4 during right rotation).

Compared with under weightbearing, the compression deformation mode of the disc changed significantly under the extra 10 kg load. Except for 1/20 of the L4–5 disc area during left rotation, the remaining discs produced similar tensile deformation in the anterior larger region (4/25–1/5 of total area). The changing trend of the L3–4 disc during right rotation under 10 kg load was comparable to that under weightbearing. However, in other cases, the compression deformation trend direction under 10 kg load was closer to the sagittal direction.

### Disc deformation at discrete locations

**Fig. 5:**
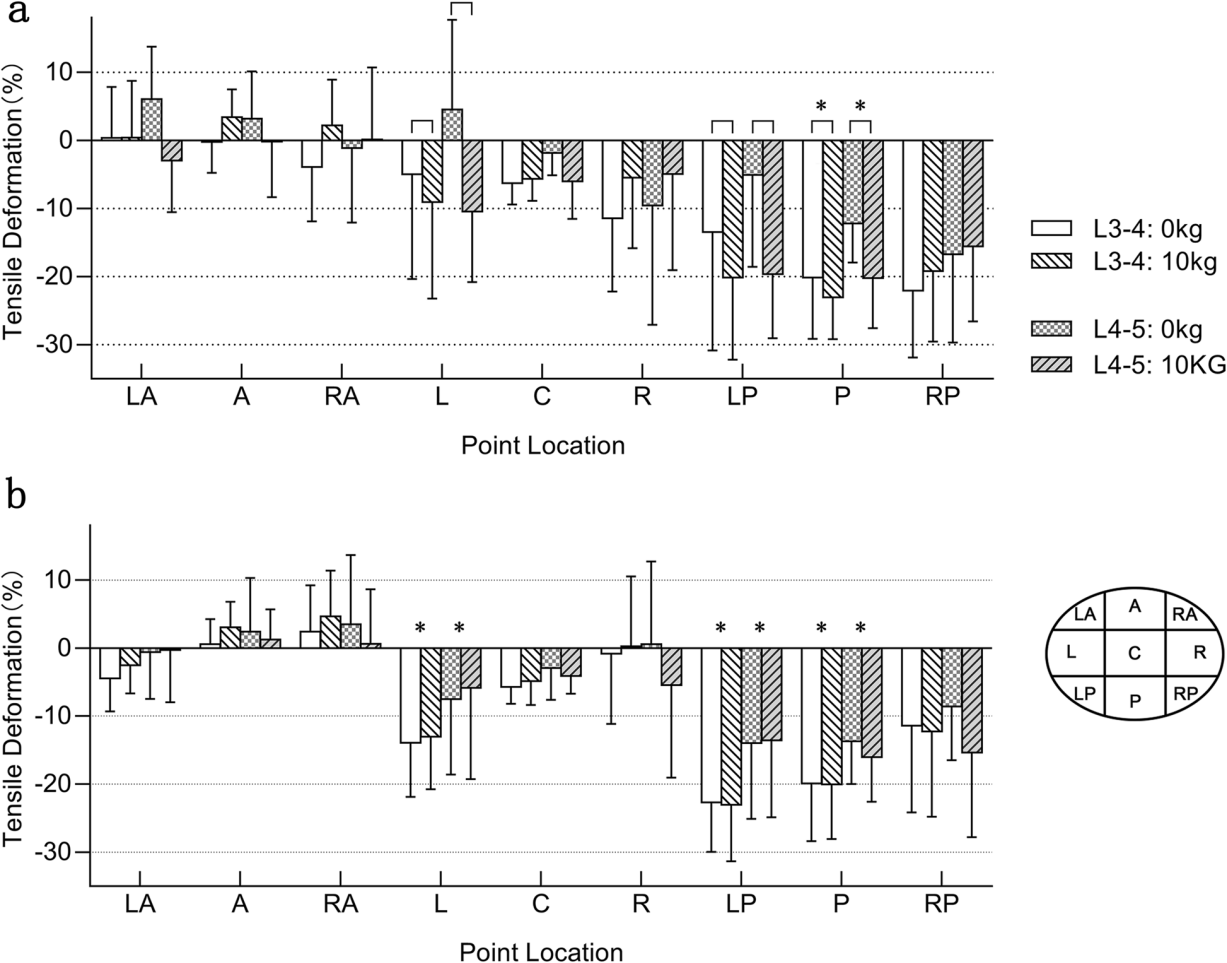
During **a** left axial rotation and **b** right axial rotation, tensile deformation at the 9 representative locations. Two-way ANOVA was used to compare the effect of segment (L3–4 and L4–5) and load (0 kg and 10 kg) on compression deformation at each point. The bars represent statistical significance upon within-level (0 kg and 10 kg) comparison of every points, whereas the symbols (*) represent statistical significance upon between level (L3–4 and L4–5) comparison of every points (*P* < 0.05). A—anterior, RA—right anterior, R—right, RP—right posterior, P—posterior, LP—left posterior, L—left, LA—left anterior and C—center

Graphs (Fig. 5) and 9-square grids (Table 1) were used to represent the quantitative data of tensile deformation at the 9 representative locations. Under a weightbearing load, the compression deformation changed diagonally from the LA to the RP during the left rotation. Symmetrically, the compression deformation changed diagonally from the RA to the LP during the right rotation. During the left rotation, the L4–5 disc exhibited a larger range of tensile deformation on the left front (LA, A and L) under weightbearing load. In the left rear (the three areas of L, LP, and P), the compression deformation of the lumbar disc under a 10 kg load was significantly greater than that under the weightbearing load. Furthermore, the compression deformation of the L3–4 disc in the posterior location was significantly greater than that of the L4–5 disc. During the right rotation, the weight load had no significant effect on the deformation of the disc. However, the deformation of the L3–4 disc in the left rear (L, LP, and P) was significantly greater than that of the L4–5 disc.

**Table 1:** 9-square grids of tensile deformation (%) at the 9 representative locations on disc surfaces

LT-0KG: L3–4			LT-10KG: L3–4		
0.37	−0.24	−3.89	0.39	3.40	2.19
−5.00	−6.26	−11.45	−9.08	− 5.65	− 5.45
− 13.44	−20.12	− 22.08	− 20.12	− 23.06	−19.18
LT-0KG: L4–5			LT-10KG: L4–5		
6.04	3.19	−1.13	−2.95	−0.18	0.15
4.55	−1.86	−9.57	−10.44	−6.01	−4.95
−5.08	−12.22	− 16.74	−19.63	−20.21	− 15.55
RT-0KG: L3–4			RT-10KG: L3–4		
−4.46	0.56	2.39	−2.54	3.01	4.64
−13.95	−5.74	−0.84	− 13.02	−4.87	0.26
−22.70	−19.91	−11.40	−23.02	− 20.04	−12.28
RT-0KG: L4–5			RT-10KG: L4–5		
−0.62	2.39	3.50	−0.27	1.22	0.62
−7.49	−2.94	0.55	−5.85	−4.10	− 5.45
−13.98	− 13.70	−8.59	− 13.59	−16.03	− 15.36

LT-Left twist rotation; RT-Right twist rotation

The shear deformation of the lumbar intervertebral disc at the 9 positions was relatively constant (Table 2). On average, the shear deformation ranged between 17 and 50%. There was no significant difference in the shear deformation under different load-bearing conditions. However, during the right rotation, the shear deformation of the lumbar disc under the weightbearing load was significantly greater than that under the 10 kg load (*P* = 0.02).

**Table 2:** Mean and standard deviation, SD, of the shear deformation (%) at the 9 representative locations

LT		LA	A	RA	L	C	R	LP	P	RP
0KG	L3–4	31.18	43.95	36.19	38.85	30.86	30.75	28.01	35.85	23.70
	SD	17.42	20.46	20.15	25.43	15.00	21.84	13.66	18.63	14.05
	L4–5	26.15	38.44	34.09	26.14	31.67	30.74	23.65	17.56	20.14
	SD	16.28	35.72	26.01	14.65	27.57	23.04	17.49	11.33	12.15
10KG	L3–4	35.49	38.60	41.00	34.64	37.40	32.73	33.10	35.14	28.96
	SD	20.02	28.69	27.42	17.77	22.54	17.89	14.77	23.81	14.83
	L4–5	40.17	43.10	42.46	41.56	40.98	35.18	39.02	32.22	23.69
	SD	24.97	27.98	27.97	31.82	25.91	26.84	30.86	27.15	13.17
RT		LA	A	RA	L	C	R	LP	P	RP
0KG	L3–4	36.03	44.96	34.96	35.68	33.61	31.10	32.41	32.01	37.19
	SD	21.38	21.37	16.35	26.11	13.88	10.61	19.02	16.65	22.17
	L4–5	26.62	42.76	50.79	27.52	34.83	42.45	17.79	32.55	26.90
	SD	19.78	32.42	30.65	17.81	30.14	31.16	18.79	26.24	17.40
10KG	L3–4	31.19	38.28	34.29	30.25	35.61	30.34	30.90	42.77	35.71
	SD	26.24	25.24	30.97	27.63	25.42	21.30	21.93	25.81	24.85
	L4–5	23.23	20.17	25.15	36.61	27.55	24.79	25.51	38.51	22.33
	SD	11.31	11.65	25.76	23.93	19.43	26.12	20.68	26.27	18.49

### Coupled bending of the lumbar spine

**Fig. 6:**
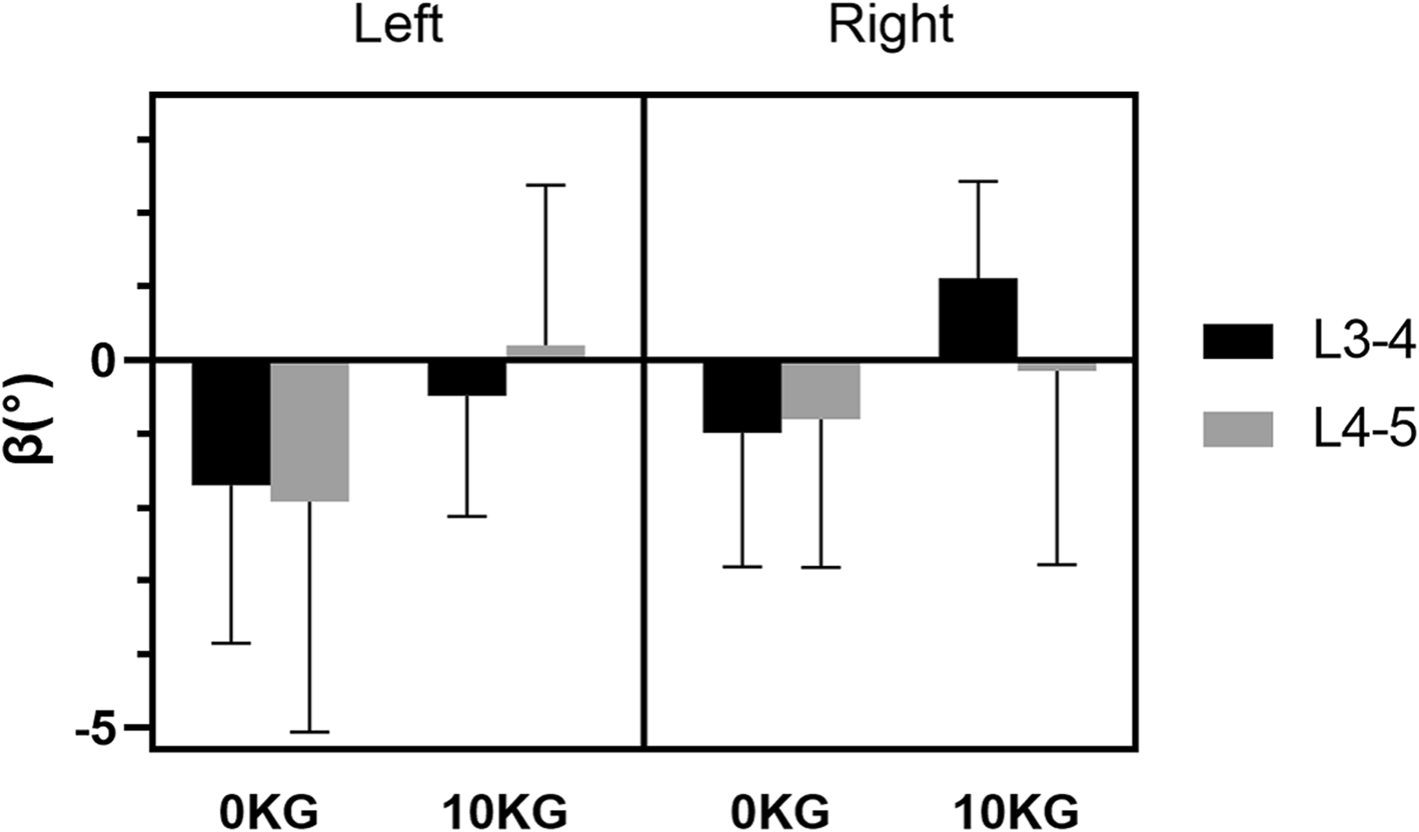
The average coupled lateral bending of the L3–5 segments during the axial rotation of the body. Error bars represent the standard deviations of the rotation range. The negative sign (−) indicated that the bending direction of the coupling was opposite to the direction of the axial rotation of the body

The range of motion (ROM) of the lumbar vertebrae during axial rotation is equal to the degree difference between maximal left-right rotation (α, β, γ, °)of each subject (Table 3). Dynamic axial rotation of the lumbar vertebrae couples flexion-extension motion and lateral bending motions. The negative sign (−) indicates that the bending direction of the coupling was opposite to the axial rotation of the body (Fig. 6) The size of the coupled bending rotation did not differ significantly between different segment levels. However, during the left rotation, the coupled lateral bending angle under the weightbearing load was significantly greater than that under the 10 kg load (*P* = 0.04).

**Table 3:** The range of motion (°) of the lumbar vertebrae at different levels during axial rotation

		Total ROM(°)	Segmental ROM(°)	
		L3–5	L3–4	L4–5
Flexion and extension (α)				
0 kg	Mean	2.38	0.94	1.44
	SD		0.48	1.18
10 kg	Mean	1.90	0.94	0.96
	SD		0.68	0.70
Left-right bending (β)				
0 kg	Mean	5.72	2.62	3.10
	SD		1.71	2.58
10 kg	Mean	3.59	1.73	1.86
	SD		1.17	1.39
Left-right axial rotations (γ)				
0 kg	Mean	2.27	1.23	1.04
	SD		0.96	0.75
10 kg	Mean	2.23	0.97	1.26
	SD		0.80	1.31

## Discussion

Rotation motion is common in a variety of swing sports and daily activities. The acquisition of quantitative data is essential for correctly understanding spine pathology and improving the surgical treatment of spine diseases. In this study, a DFIS was used to investigate the in vivo deformation data of the lumbar disc during axial rotation and compare the effects of different weight loads. Other studies have used this method to measure disc deformation data in the standing position [9].

Our data showed that, relative to the supine position, the average compression deformation caused by rotation motion was between + 10% and − 40% and the shear deformation was between 17 and 50%. Under physiological weightbearing, different levels of lumbar discs exhibited similar deformation patterns, and the left and right rotation deformation patterns were approximately symmetrical. Among them, the posterior side opposite the rotational motion experienced the largest compression deformation. Lower discs had a larger area of tensile deformation than upper discs on the anterior of the same side as the direction of rotational motion. It was also found that the deformation pattern changed significantly under a 10 kg load, with the exception of the L3–4 disc during the right rotation. The disc deformation changed from front to back, as opposed to the diagonal change under weightbearing. The shear deformation range was relatively constant, and no obvious regularity was found.

**Fig. 7:**
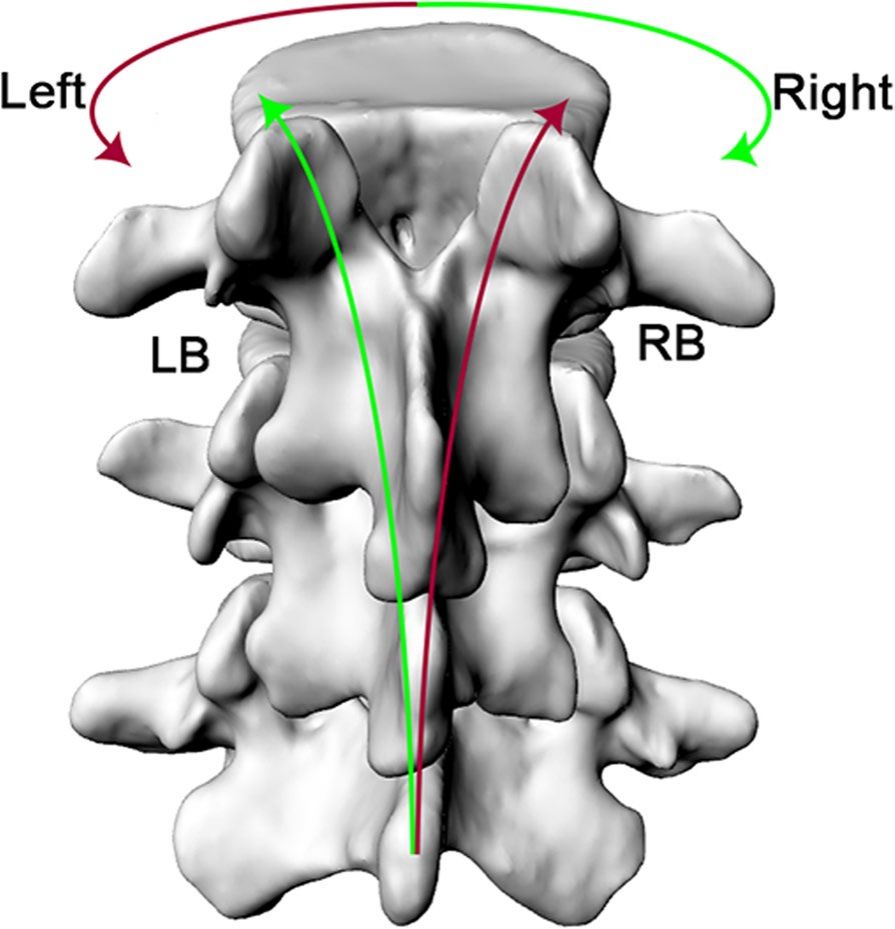
Rotational movement coupled with lateral bending in opposite directions: LB-left bending, RB-right bending

A dynamic axial rotation of the body is complicated and needs a coordinated dynamic coupling of flexion-extension, left-right bending, and left-right axial rotations of the lumbar vertebrae to maintain the global dynamic balance of the body. While the facet joints bear the compressive load of the spine, the facet morphology restricts the movement of the vertebral body. Quantitative knowledge of lumbar kinematics during axial rotation has been investigated in various in vitro and in vivo studies. Li et al. [13] found that the axial rotation ranges were 2.4 ± 2.6° for L3–4 and 2.9 ± 2.1° for L4–5 and that the coupled bending rotation was between 2.0° and 3.0° using DFIS. Shin et al. [14] further investigated the relationship between primary axial rotation and coupled bending. The results were partially consistent with previous research. These studies have found that the L3–4 segment coupled bent in the opposite direction of the axial rotation. However, the controversy mainly focused on the L4–5 segment. Panjabi et al. and Shin et al. showed that the side bending motion was in the same direction as the rotation [14, 17]. Ochia et al. and Fujii et al. found that the side bending motion was opposite to the rotation motion direction, which is similar to our data [5, 18] (Fig. 7). These differences could be explained by their respective movement patterns. In our study, subjects performed voluntary exercises that were guided and restricted to some extent.

These studies mainly focused on the ROM of rotational motion and rarely considered disc deformation. There have been many studies focusing on stress changes in the disc, mainly in the standing position and during flexion-extension motion. Wang et al. [9] measured the geometric deformation of the lumbar disc under physiological weightbearing. To keep body weight balanced while standing, lumbar lordosis increased, causing the front part of the disc to tensile and the back part to compress. This explained the deformation pattern of the lumbar disc during rotation. As the body moves from the supine to the standing position and then to axial rotation, the combined factors of the load and coupled lateral bending cause diagonal deformation. Research has shown that the disc is subjected to significantly stronger compressive loads in the posterolateral direction during axial rotation, which may a risk factor for disc degeneration. In addition, these data serve as a foundation for the design of artificial discs that can better adapt to changes in the human body’s different motion stresses.

In our study, under a 10 kg load, the coupled bending of the lumbar spine was significantly reduced, resulting in a change in the deformation pattern. The coupled lateral bending assisted in maintaining body balance during body axial rotation, but it may be affected by the extra weight. The combination of extra weight and axial rotation causes the lumbar spine to endure a bending moment during axial rotation, which may lead to an increase in muscle strength and changes in facet joint stress, thus changing the biomechanics of the lumbar spine. This discovery of the changes in disc stress during in vivo loading movement may be vital; it provides a new perspective on the model of three-pillar vertebral segment degeneration after long-term loading.

The study has several limitations. Because of small sample size, our observation ability is limited, resulting in some differences that are not statistically significant and a large SD. In addition, due to the size limitations of the fluoroscope and the influence of the machine, we only checked the end-of-motion state of the L3–5 disc and did not analyze the instantaneous position. Despite these limitations, we measured the disc deformation pattern in conjunction with rotation and bending from a new perspective. Future research should include more segments and weightbearing loads.

In summary, we found that the deformation of the lumbar disc exhibited direction specificity and level specificity during axial rotation, which was coupled with lateral bending movement to maintain body balance, which can be affected by weight bearing. These findings can provide new insights into the biomechanics of the human lumbar spine and references for finite element and degeneration studies.

## Conclusion

The deformation of the lumbar disc was direction-specific and level-specific during axial rotations and was affected by extra weight. These data can provide new insights into the biomechanics of the lumbar spine and optimize the parameters of artificial lumbar spine devices.

## Data Availability

The datasets used and/or analyzed during the current study are available from the corresponding author on reasonable request.

## Abbreviation

DFIS: Dual Fluoroscopic Imaging System
