# Correction: Investigation of geometric deformations of the lumbar disc during axial body rotations

**DOI:** 10.1186/s12891-022-05654-6

**Published:** 2022-08-09

**Authors:** Haoxiang Xu, Wangqiang Wen, Zepei Zhang, Jianqiang Bai, Bowen Kou, Jun Miao

**Affiliations:** 1 Clinical Department of Orthopaedics, Tianjin Medical University, Tianjin, China; 2 Department of Spine Surgery, Tianjin Hospital, Jiefangnanlu 406, Hexi District, Tianjin, China

**Correction: BMC Musculoskelet Disord 23, 225 (2022)**

**https://doi.org/10.1186/s12891-022-05160-9**

Following the publication of the original article [1], the authors found the incorrectly direction of coupled lateral bending under 10 kg load during right rotation in Fig. 6 and herein make corrections of these errors. The erratum includes the new Fig. 6.

**Fig. 6:**
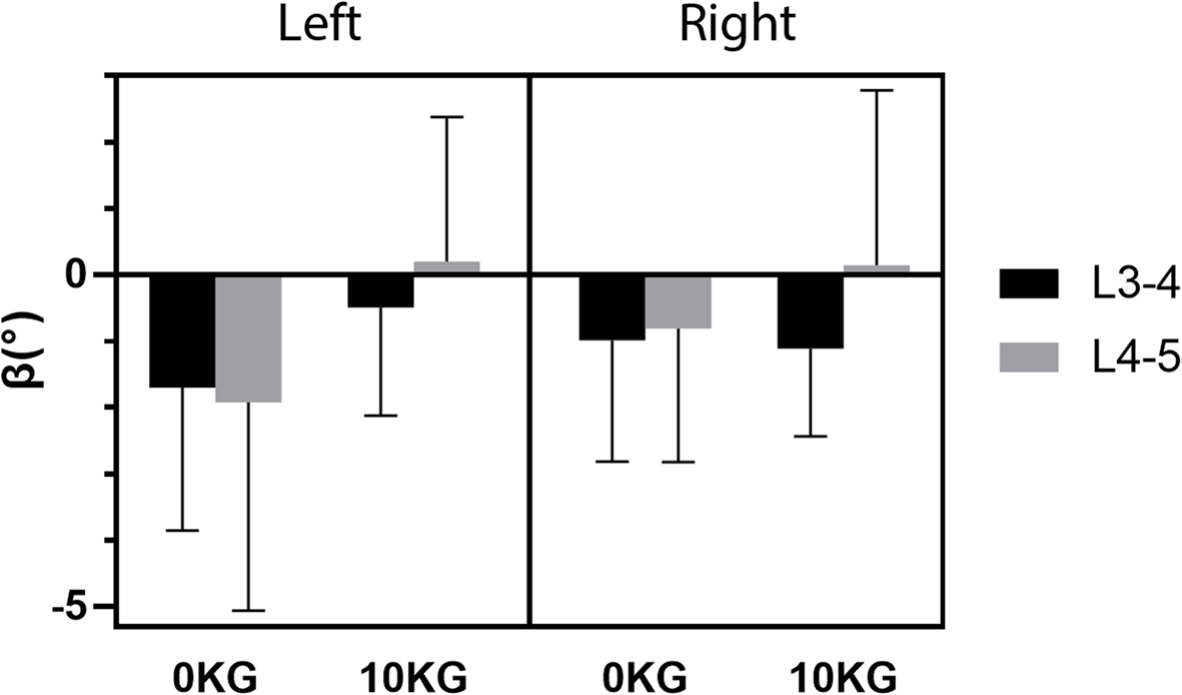
The average coupled lateral bending of the L3–5 segments during the axial rotation of the body. Error bars represent the standard deviations of the rotation range. The negative sign (−) indicated that the bending direction of the coupling was opposite to the direction of the axial rotation of the body

The original article [1] has been updated.

## References

[1] Xu H, Wen W, Zhang Z (2022). Investigation of geometric deformations of the lumbar disc during axial body rotations. BMC Musculoskelet Disord.

